# Gastric hydrodistension CT versus CT without gastric distension in preoperative TN staging of gastric carcinoma: analysis of single-center cancer registry

**DOI:** 10.1038/s41598-022-15619-3

**Published:** 2022-07-05

**Authors:** Yu-Hsien Lee, Wen-Hui Chan, Ying-Chieh Lai, An-Hsin Chen, Chien-Ming Chen

**Affiliations:** Department of Medical Imaging and Intervention, Chang Gung Memorial Hospital at Linkou, and Chang Gung University College of Medicine, 5 Fuxing Street, Guishan District, Taoyuan, Taiwan

**Keywords:** Cancer imaging, Gastrointestinal cancer

## Abstract

Accurate staging of gastric cancer is essential for the selection and optimization of therapy. Hydrodistension of the stomach is recommended to improve the accuracy of preoperative staging with contrast-enhanced multidetector computed tomography (MDCT). This study compares the performance of contrast-enhanced gastric water distension versus a nondistension MDCT protocol for T and N staging and serosal invasion in comparison to surgical histopathology. After propensity score matching, 86 patients in each group were included for analysis. The overall accuracy of distension versus nondistension group in T staging was 45% (95% CI 35–56) and 55% (95% CI 44–65), respectively (*p* = 0.29). There was no difference in the sensitivity and specificity in individual T staging and assessment of serosal invasion (all *p* > 0.41). Individual stage concordance with pathology was not significantly different (all *p* > 0.41). The overall accuracy of N staging was the same for distension and nondistension groups (51% [95% CI 40–62]). The majority of N0 staging (78–81%) were correctly staged, whereas N3 staging cases (63–68%) were predominantly understaged. In summary, there was no significant difference in the diagnostic performance of individual TN staging and assessment of serosal invasion using MDCT with or without gastric water distension.

## Introduction

Gastric cancer is an important cause of cancer-related death in East Asia, South America and Eastern Europe^1^. While screening in high incidence areas such as Japan and Korea has led to reductions in gastric cancer-associated mortality^2,3^, surgical or endoscopic resection remains the backbone of curative intent treatment^4–6^. Despite recent treatment strategies such as neoadjuvant and adjuvant chemotherapy, the overall 5-year survival rate remains poor (35–45%)^7,8^.

Accurate staging of gastric cancer is essential for the selection and optimization of therapy. After initial diagnosis by gastroscopy, diagnostic work-up can consist of endoscopic ultrasonography (EUS) or contrast-enhanced multidetector computed tomography (MDCT)^9^. A recent meta-analysis consisting of eight studies (*n* = 1736) compared EUS to MDCT and showed that EUS had higher sensitivity than MDCT (82% versus 41%; *p* = 0.03) in detecting T1 staging^10^. However, no significant differences in T2–4 staging were noted between EUS and MDCT. Distension of the stomach with air and/or water to better visualize interruptions to the multilayered pattern of the normal gastric wall has been recommended to improve the accuracy of preoperative staging of gastric cancer through multiplanar reconstructions and virtual gastroscopy^11–18^. However, such accuracy studies have been limited by small sample sizes^19–21^, patient selection^15,19,20^, imbalances in the number of patients in each T staging^15,19,21^, and the lack of head-to-head comparisons with the nondistended stomach on modern MDCT^11–13^. A recent clinical audit of the authors’ hospital cancer registry sought to enumerate the local diagnostic performance of MDCT for gastric cancer staging.

The purpose of this single institution cancer registry study is twofold: first, to analyze the preoperative T and N staging performance of a dedicated water-distended gastric MDCT protocol versus a nondistended MDCT protocol; second, to evaluate the performance of the two protocols in the detection of serosal invasion.

## Results

### Study cohort

In total 1156 patient records were reviewed, and a final cohort of 516 patients was included after the following exclusions: clinical metastasis at presentation (*n* = 340), pathological metastasis at surgery (*n* = 19), no surgical histopathology (*n* = 55), nonadenocarcinoma histopathology (*n* = 53), CT performed at referral hospital (*n* = 125), and CT performed at the emergency department (*n* = 48).

### Propensity score matching

Table 1 tabulates the characteristics of the cohort study at baseline and after propensity score matching. There were class imbalances in the age, sex, origin of CT, pT staging, and pN staging at baseline. The baseline cohort was mostly male (61%, 314/516), with cancers that occurred in the antrum (53.5%, 276/516) and had CT performed as inpatients (74%, 381/516). Early gastric cancer (T1 staging) accounted for 33% (168/516). In the postmatched cohort, both Distension and Nondistension groups consisted of 86 patients each, with 50% (86/172) of cases occurring in the antrum and 32% (55/172) of cases of T1 staging, similar to the baseline cohort.

**Table 1:** Patient characteristics before and after propensity score matching.

Covariate	Pre-match			Post-match		
	Distension (*n* = 402)	Non-distension (*n* = 114)	SMD	Distension (*n* = 86)	Non-distension (*n* = 86)	SMD
Age (year)	65 (55–74)	68 (57–76)	0.19	68 (58–77)	66 (54–75)	− 0.01
**Sex**			0.18			0.07
Male	237 (59.0)	77 (67.5)		53 (61.6)	56 (65.1)	
Female	165 (41.0)	37 (32.5)		33 (38.4)	30 (34.9)	
**Origin of CT**			0.37			0.01
Inpatient	300 (74.6)	81 (71.1)		62 (72.1)	44 (51.2)	
Outpatient	102 (25.4)	33 (28.9)		24 (27.9)	33 (48.8)	
**Year of diagnosis**			− 0.12			− 0.18
2010	76 (18.9)	26 (22.8)		22 (25.6)	23 (26.7)	
2011	76 (18.9)	21 (18.4)		11 (12.8)	14 (16.3)	
2012	89 (22.1)	21 (18.4)		25 (29.1)	16 (18.6)	
2013	78 (19.4)	33 (28.9)		12 (14.0)	23 (26.7)	
2014	83 (20.6)	13 (11.4)		16 (18.6)	10 (11.6)	
**Site of tumor**			0.06			− 0.18
Fundus	36 (9.0)	17 (14.9)		10 (11.6)	15 (17.4)	
Body	151 (37.6)	26 (22.8)		33 (38.4)	23 (26.7)	
Antrum	209 (52.0)	67 (58.8)		41 (47.7)	45 (52.3)	
Diffuse	6 ((1.5)	4 (3.5)		2 (2.3)	3 (3.5)	
**Laurens**			− 0.08			0.05
Intestinal	166 (41.3)	50 (43.9)		34 (39.5)	35 (40.7)	
Diffuse	149 (37.1)	43 (37.7)		35 (40.7)	34 (39.5)	
Mixed	87 (26.1)	21 (18.4)		17 (19.8)	17 (19.8)	
**pT staging**			0.31			− 0.14
1	144 (35.8)	24 (21.1)		32 (37.2)	23 (26.7)	
2	35 (8.8)	14 (12.3)		5 (5.8)	12 (14.0)	
3	133 (33.1)	40 (35.1)		29 (33.7)	32 (37.2)	
4a	66 (16.4)	26 (22.8)		12 (14.0)	14 (16.3)	
4b	24 (6.0)	10 (8.8)		8 (9.3)	5 (5.8)	
**pN staging**			0.23			− 0.03
0	159 (39.6)	36 (31.6)		32 (37.2)	32 (42.1)	
1	60 (14.9)	13 (11.4)		11 (12.8)	12 (2.6)	
2	81 (20.1)	26 (22.8)		16 (18.6)	17 (22.4)	
3	102 (25.4)	39 (34.2)		27 (31.4)	25 (32.9)	

*SMD* standardized mean difference.

SMD of greater than 0.1 is significant. Age given in median with interquartile range in parenthesis. Data presented as counts and percentages in parenthesis.

### Diagnostic performance

Interrater reliability for the independent readers is shown in Table 2. In both Distension and Nondistension groups, the ICC was good to excellent for T and N staging, indicating that readers had a high degree of agreement.

**Table 2:** Inter-rater reliability.

	Distension	Non-distension
T staging	0.90 (0.86–0.93)	0.90 (0.84–0.94)
N staging	0.90 (0.86–0.93)	0.92 (0.89–0.94)

Intraclass correlation coefficients (ICC) presented with 95% confidence intervals in parenthesis is based on mean-rating, absolute-agreement, 2-way random effects model. For TN staging ICC based on 4 independent readers and 1 tumor board reading.

**Figure 1:**
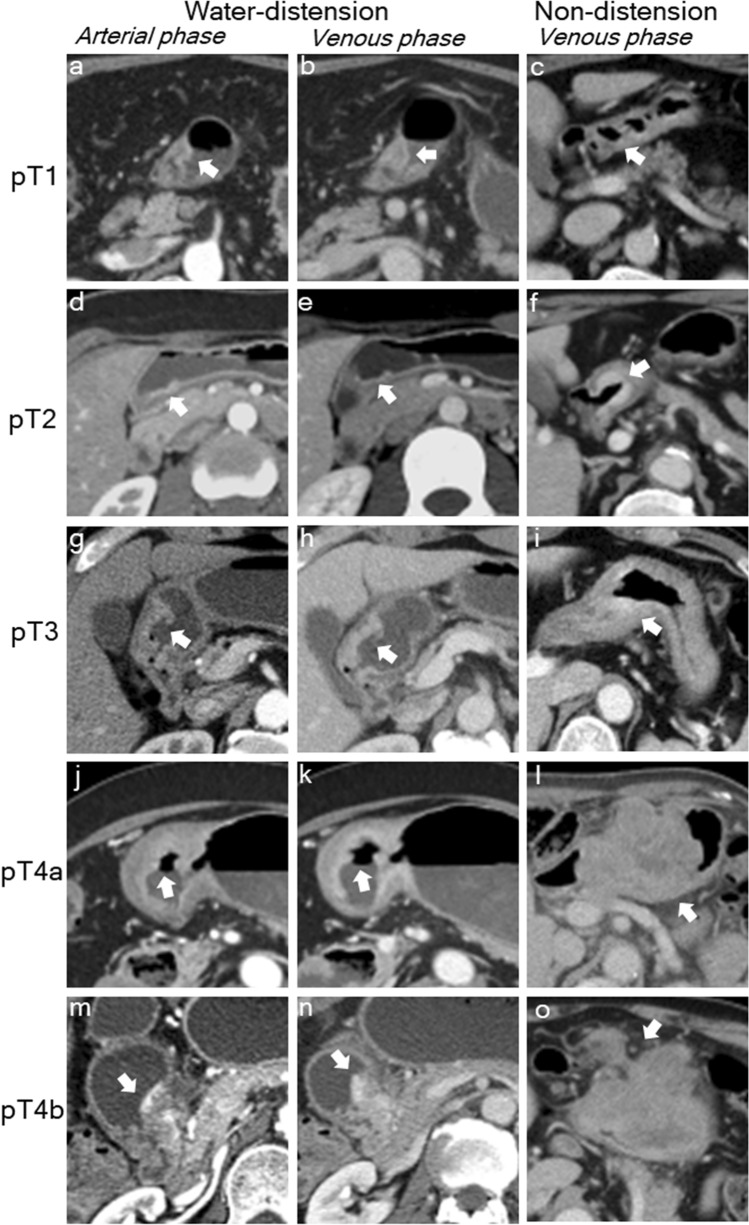
Clinical images of water-distension versus nondistension MDCT protocols. The water-distension protocol is shown with arterial (**a**,**d**,**g**,**j**,**m**) and venous (**b**,**e**,**h**,**k**,**n**) phase images. Nondistension protocol is shown with venous phase (**c**,**f**,**i**,**l**,**o**). The white arrow points to the primary gastric cancer. In pT4b stage cancers, the invasion of the pancreas (1**n**) and transverse colon (1**o**) is noted.

Table 3 tabulates the T and N staging performance metrics of Distension and Nondistension groups derived from consensus reading. The overall accuracy of Distension versus Nondistension groups in T staging was 45% (95% CI 35–56) and 55% (95% CI 44–65), respectively (*p* = 0.29), and there was no difference in the overall accuracy for N staging in either group (51% [95% CI 40–62] in both). There were no significant differences in sensitivity and specificity of individual T and N staging between Distension and Nondistension group. Figure 1 shows clinical case examples of T1 to T4b staging cancers in Distention and Nondistention groups.

**Table 3:** Diagnostic performance of individual TN staging.

Staging	Distension (*n* = 86)					Nondistension (*n* = 86)					*p* sensitivity	*p* specificity
	Sensitivity (%)	Specificity (%)	PPV (%)	NPV (%)	Overall accuracy (%)	Sensitivity (%)	Specificity (%)	PPV (%)	NPV (%)	Overall accuracy (%)		
**T staging**					45 (35–56)					55 (44–65)		
T1	34 (19–53)	100 (93–100)	100 (72–100)	72 (60–82)		48 (27–69)	100 (94–100)	100 (72–100)	84 (74–91)		0.41	1
T2	60 (15–95)	74 (63–83)	12 (3–32)	97 (89–100)		67 (35–90)	86 (77–93)	44 (22–69)	94 (86–98)		1	0.07
T3	59 (39–76)	79 (66–89)	59 (39–76)	79 (66 -89)		56 (38–74)	74 (60–85)	56 (38–74)	74 (60–85)		1	0.66
T4a	33 (10–65)	85 (75–92)	27 (8–55)	89 (79 -95)		43 (18–71)	85 (74–92)	35 (14–62)	88 (78–95)		0.70	1
T4b	50 (16–84)	96 (89–99)	57 (18–90)	95 (88 -99)		80 (28–99)	95 (88–99)	50 (16–84)	99 (93–100)		0.57	1
**N staging**					51 (40–62)					51 (40–62)		
N0	78 (60–91)	70 (56–82)	61 (45–76)	84 (71–94)		81 (64–93)	65 (51–77)	58 (42–72)	85 (71–94)		1	0.68
N1	0 (0–28)	85 (75–92)	0 (0–28)	85 (75–92)		25 (5–57)	86 (77–93)	23 (5–54)	88 (78–94)		0.22	1
N2	56 (30–80)	83 (72–91)	43 (22–66)	89 (79–96)		41 (18–67)	84 (73–92)	39 (17–64)	85 (75–93)		0.49	1
N3	37 (19–58)	95 (86–99)	77 (46–95)	77 (65–86)		32 (15–54)	97 (89–100)	80 (44–97)	78 (67–86)		0.78	0.68

Data presented as percentages and 95% confidence interval in parenthesis.

*NPV* negative predictive value, *PPV* positive predictive value.

Table 4 compares the diagnostic performance for assessing serosal invasion in Distension and Nondistension groups. The sensitivity was greater than 90%, and the positive predictive value greater than 80% in both groups. However, no significant difference in diagnostic performance was demonstrated between Distension and Nondistension groups.

**Table 4:** Comparison of diagnostic performance for serosal invasion.

	Distension	Non-distension	*p*
Sensitivity (%)	90 (78—97)	94 (84—99)	0.48
Specificity (%)	81 (65—92)	74 (57—88)	0.58
Positive predictive value (%)	86 (74—94)	84 (72—93)	0.89
Negative predictive value (%)	86 (70—95)	90 (73—98)	0.86

Data presented as percentages and range in parenthesis.

Discrepancies in clinical and pathological TN staging are shown in Table 5. Concordant staging for T2, T3, T4a and T4b was 60%, 59%, 33% and 50%, respectively, for the Distension group and 67%, 58%, 43%, 80%, respectively, for the Nondistension group. In overstaged pT1 lesions, 24% (5/21) in Distension group and 42% (5/12) in Nondistension group were overstaged by two levels (*p* = 0.48). There was no significant difference in each individual T or N staging between the two groups (all *p* > 0.40).

**Table 5:** Clinical versus pathological TN staging concordance.

	Distension (*n* = 86)			Nondistension (*n* = 86)			*p*
	Under stage	Correct stage	Over stage	Under stage	Correct stage	Over stage	
pT1		11 (34)	21 (66)^a^		11 (48)	12 (52)^a^	0.41
pT2		3 (60)	2 (40)		8 (67)	4 (33)	1
pT3	3 (10)	17 (59)	9 (31)	2 (6)	18 (58)	12 (38)	0.80
pT4a	8 (67)	4 (33)		6 (43)	6 (43)	2 (14)	0.44
pT4b	4 (50)	4 (50)		1 (20)	4 (80)		0.57
pN0		25 (78)	7 (22)		26 (81)	6 (19)	1
pN1	7 (64)	0 (0)	4 (36)	7 (58)	3 (25)	2 (17)	0.25
pN2	7 (44)	9 (56)		9 (53)	7 (41)	1 (6)	0.61
pN3	17 (63)	10 (37)		17 (68)	8 (32)		0.78

Data presented as counts and percentages in parenthesis.

^a^In over staged pT1 lesions, 24% (5/21) in Distension group and 42% (5/12) in Nondistension group were over staged by two levels (*p* = 0.48).

The majority of N0 staging cases were correctly staged, whereas the N3 staging cases were predominantly understaged. This is also reflected in the higher sensitivity of N0 staging in both groups (Distension 78% versus Nondistension 81%, *p* = 1).

## Discussion

This study showed that the overall accuracy of T and N staging, sensitivity and specificity for the identification of individual T and N staging for gastric cancer with MDCT was not significantly different with or without water-distension. The Nondistension group showed a trend toward higher sensitivity than Distension group at each individual T staging (Distension versus Nondistension T1, T2, T3, T4: 34%, 60%, 59%, 55% versus 48%, 67%, 56%, 67%; *p* > 0.41). This result is in keeping with findings from a recent meta-analysis showing pooled sensitivities of 41%, 48%, 64%, and 61% for T1, T2, T3 and T4 staging respectively^10^. Sensitivity (Distension versus Nondistension, 90% [95% CI 78–97] versus 94% [84–99]) and specificity (81% [65–92] versus 74% [57–88]) in assessing serosal invasion was not significantly different between groups (*p* = 0.48 and *p* = 0.58, respectively). This contrasts with results reported by Hasegawa et al. (using 7 mm axial slices), who showed a lower sensitivity of 55% (43–66), but a higher specificity of 94% (90–96)^22^.

Clinical T staging concordance with pathology enables proper selection of patients for neoadjuvant chemotherapy. Despite gastric distension with water, stage concordance in the Distension group was all below 60%. There was, however, a trend toward higher stage concordance in the Nondistension group. In a Norwegian study on restaging CT following neoadjuvant chemotherapy, only 24% had stage concordance, 38% overstaged and 38% understaged (kappa = 0.06 [95% CI 0.004–0.12])^23^. In the Japanese JCOG1302A trial, stage concordance for pT2, pT3, pT4a and pT4b was 40% (94/235), 45% (184/407), 55% (209/380) and 4% (1/25) respectively^24^. In another series at Shizuoka Cancer Center in Japan, stage concordance for pT1, pT2, pT3 and pT4 was 89% (1951/2197), 25% (133/530), 36% (106/294) and 58% (622/1080) respectively^25^.

The European MAGIC trial on perioperative chemotherapy found that 8.3% of patients who underwent gastrectomy alone had pT1 disease^7^. In the present study, 24% (5/21) of the Distension group and 42% (5/12) of the Nondistension group overstaged pT1 as greater than equal to T3 staging (*p* = 0.48). In a Korean series with modern 64-dectector CT and where 60% (76/127) of lesions were pT1 (due to an extensive country-wide screening program), the mean stage concordance for pT1, pT2, pT3 and pT4a was 97%, 66%, 70%, and 80%, respectively^26^. As in most series, the higher stage concordance for pT1 may be because the absence of lesions on MDCT in a known gastric cancer patient is often diagnostic for pT1 staging^15,27,28^.

In comparing pT3-4 staging accuracy in the JCOG1302A trial, stage concordance was similar for MDCT with 1-mm or 5-mm slice thickness (44% [37–50] versus 42% [39–45]), but worse for MDCT with gastric air distension (39% [34–44] versus 45% [42–48])^29^. The sensitivity was higher for 1-mm versus 5-mm (90% [84–94] versus 84% [82–87]) and higher for CT with air distension (87% [82–91] versus 84% [81–87]). Overall, there were no remarkable differences in the comparisons. Of note, in this trial, more than 2 times the number of CTs performed was without air distension than with air distension. Our findings are in keeping with the JCOG1302A trial; gastric distension for pT3–4 lesions did not improve staging concordance (all *p* > 0.44). Indeed, the classic three-layered pattern of the gastric wall in contrast-enhanced CT images^11,26,30^, which is critical for the evaluation of T1–2 lesions and accentuated with gastric distension, is not applicable in T3–4 lesions.

The diagnostic performance of N staging was not affected with or without gastric distension with water. Indeed except for N0 staging (> 78%), N staging sensitivity was uniformly poor (< 60%). Size (> 8 or > 10 mm), morphology, clustering and enhancement are often used to determine whether nodal metastasis is present, but invariably, small lymph nodes may be sites of metastasis. The literature reported a sensitivity ranging from 4 to 17% for T1 tumors^31,32^ to 63% for T2–4 tumors^33^ and 67–87% when T1–4 tumors are considered^34–36^. The use of PET/CT does not improve upon sensitivity of N staging (38–40%)^37,38^. In one series, the N staging concordance was 73% (2244/3066), 29% (105/368), 23% (79/348) and 72% (97/134) for N0, N1, N2, and N3 respectively^25^. In the JCOG1302A trial, the N0 staging concordance was 66% (278/423)^24^. In the current study, pN0 staging concordance was 78% (25/32) and 81% (26/32) for Distension and Nondistension groups, respectively. The majority of pN1–3 staging in both groups was understaged (48–68%).

There are a few limitations in this study. First, the water-distension protocol involved supine-only imaging irrespective of the location of the tumor. In this study, antrum and body tumors accounted for more than three quarters of all tumors. Second, virtual gastroscopy or tailored multiplanar reconstruction relative to the primary lesion was not performed as these techniques required additional reading and time at the workstation; this is not part of the standard workflow in our institution. Third, pT1 accounted for one-third of cases, which reflects the real-world prevalence in our population. While EUS staging may be preferable and more sensitive for T1 lesions, MDCT still forms an important part of preoperative staging. Finally, the independent readers were aware of the location of the tumor identified by endoscopy, this may have affected the diagnostic performance. However, in routine clinical practice, final clinical staging is performed by members of the gastric cancer multidisciplinary tumor board with access to endoscopic images for review.

In conclusion, the overall accuracy, individual T and N staging sensitivity and specificity of MDCT with or without gastric distension with water were not significantly different. The sensitivity and specificity in the detection of serosal invasion on MDCT were not affected with or without gastric distension with water.

## Material and methods

### Study design and study cohort

This was a single institution retrospective review of the gastric cancer registry from 2010 to 2015, which encompasses the 7th edition of the American Joint Committee on Cancer (AJCC)/Union for International Cancer Control (UICC) staging system. Patient demographics, origin of CT image (inpatient, outpatient, outside referral, and emergency department), site of tumor (antrum, body, fundus or diffuse [greater than two contiguous sites]), clinical (based on the initial CT stage from multidisciplinary tumor board) and pathological TNM stage, histopathological subtype based on WHO and Lauren’s classification (intestinal, diffuse, and mixed), and the CT protocol were evaluated.

**Figure 2:**
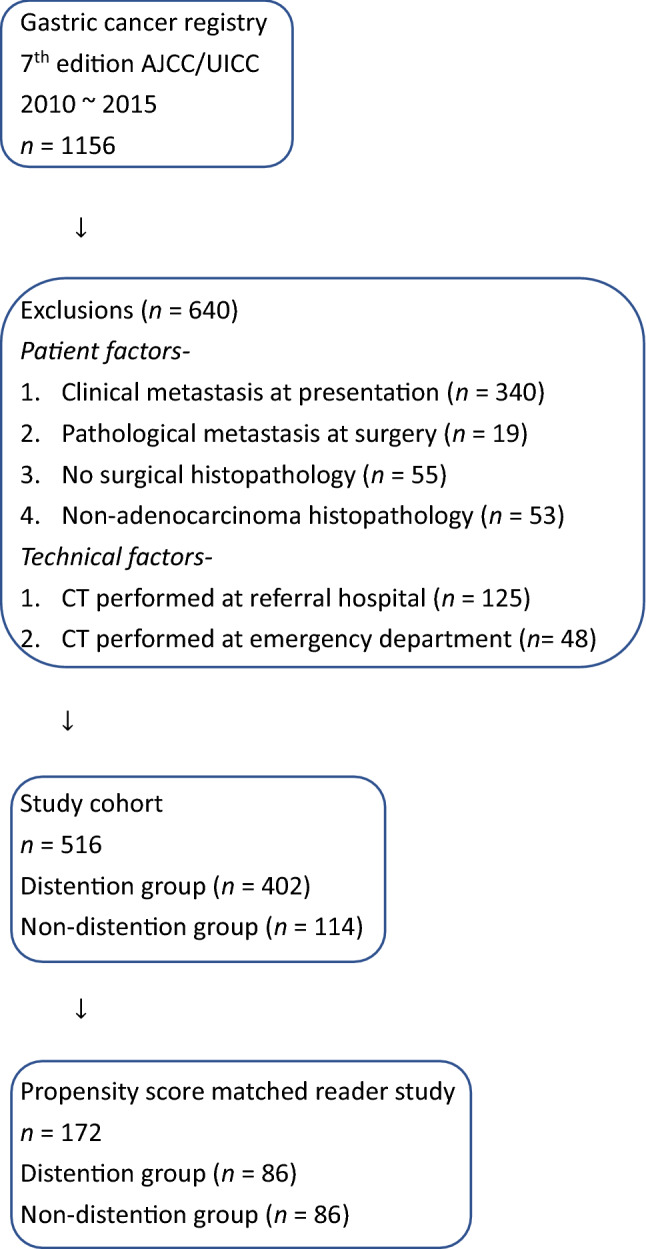
Flowchart of the study design.

The registry includes all patients who were diagnosed or referred to our hospital for the treatment of gastric cancer. The following exclusion criteria were applied: clinical metastasis at presentation, pathological metastasis at surgery, no surgical histopathology, nonadenocarcinoma histopathology, CT performed at the referral hospital, and CT performed at the emergency department. CT images from referral hospitals and from the emergency department were excluded because of inconsistent image quality and/or reconstruction parameters. The study design flowchart is shown in Fig. 2.

We sought to compare the diagnostic performance of two CT protocols (described in detail below) in the evaluation of T and N staging to that of surgically-resected specimens. This study was approved by the Chang Gung Medical Foundation Institutional Review Board and a waiver of informed consent was approved as the research involves no more than minimal risk to subjects. All study methods were carried out in accordance with relevant guidelines and regulations. The STARD guidelines were used to ensure the reporting of this diagnostic accuracy study^39^.

### CT protocols

Owing to the reimbursement policy of the national health care system, patients whose CT was performed at the time of presentation had restricted access to secondary dedicated staging CT after the diagnosis of gastric cancer. In this group of patients, gastric distension was not performed prior to CT acquisition. For the purpose of this study, we defined two types of protocols: a water-distension dual-phase protocol for staging of newly diagnosed gastric cancer (hereafter referred to as “Distension”), and a nonwater distended (“Nondistension”) protocol.

Per the Distension protocol, the patient ingest up to 1000 mL of purified water prior to CT examination to distend the stomach. Scan acquisition was supine-only covering the whole stomach during arterial phase and the abdomen to the pelvis during the venous phase. Axial, coronal and sagittal arterial phase images were routinely reconstructed into 3 mm slice thickness and interval; axial venous phase images were reconstructed into 5 mm slice thickness and interval.

Protocols designated as Nondistension included contrast-enhanced single and dual-phase CT of the abdomen and pelvis without prior distension of the stomach. For this protocol, axial and coronal images from venous phase images were reconstructed into 5 mm thickness and interval. All CT examinations were performed with 16- or 64-detector CT scanners.

### Propensity score matching

The study cohort consisted of imbalanced groups (Distension versus Nondistension 402:114). Propensity-score matching was performed logistic regression model with the following covariates: age, sex, pT staging, pN staging, Lauren’s classification, tumor site, CT origin, year of diagnosis. The standardized mean difference was used to evaluate matching between groups, with a value of 0.1 or higher indicating an imbalance. Propensity score matching was performed using R version 4.0.2 (R Foundation for Statistical Computing, Vienna, Austria) with the MatchIt package (version 3.0.2)^40^ using the nearest neighbor matching method and a caliper distance of 0.2 without replacement.

### Secondary image and tumor stage evaluation by independent readers

Over the 5-year study period, different and/or additional radiologists joined the gastric cancer multidisciplinary tumor board and rendered the clinical TNM stage. To mitigate bias and evaluate the reliability and reproducibility of image readings by different radiologists, additional image review by 4 radiologists who were members of the gastric cancer multidisciplinary tumor board (C.M.C, W.H.C, Y.S.L, Y.C.L with 11, 7, 7, and 3 years of experience, respectively) was performed. The clinical TN stage established during the tumor board conference was included as a 5th independent reader. All readers performed the image interpretation separately and were independently blinded to the pathological TN staging. Images from the same CT protocol were batched and then randomized for reading on two separate occasions. Readers noted the clinical T (1, 2, 3, 4a and 4b), and clinical N (0, 1, 2, 3) staging based on the AJCC/UICC 7th edition.

### Statistical analysis

Continuous variables are presented as the means ± standard deviation or, when the distributions are skewed, as the medians and interquartile ranges. Categorical variables were compared using the Chi-Square test or Fisher’s Exact test. In the independent reader study, interrater reliability with intraclass correlation coefficient (ICC) estimates and their 95% confident intervals were calculated using R with the irr package (version 0.84.1) based on a mean-rating (k = 5), absolute-agreement, 2-way random effects model. Intraclass correlation coefficient values less than 0.5 were indicative of poor reliability, values between 0.5 and 0.75 indicated moderate reliability, values between 0.75 and 0.9 indicated good reliability, and values greater than 0.9 indicate excellent reliability^41^. Consensus TN staging was calculated from the mode (majority consensus) of all 5 reader observations. Diagnostic performance (sensitivity, specificity, positive predictive value, negative predictive value) with 95% confidence intervals was calculated from the consensus TN staging. Clinical versus pathological TN staging concordance (under stage, correct stage, over stage) was calculated. The statistical significance was set at *p* < 0.05.

## Data Availability

The datasets used and/or analysed during the current study available from the corresponding author on reasonable request.
